# A Flowable Placental Tissue Matrix Allograft in Lower Extremity Injuries: A Pilot Study

**DOI:** 10.7759/cureus.275

**Published:** 2015-06-10

**Authors:** Eric Lullove

**Affiliations:** 1 West Boca Medical Center, Boca Raton, FL

**Keywords:** sports injuries, amniotic allograft, injectables, allografts, human placenta, sports medicine

## Abstract

Damaged connective tissue commonly leads to lower extremity injuries. These injuries can result in inflammation, reduced mobility, and chronic pain. Conservative treatment may include orthotics, offloading the injury, physical therapy, and/or NSAIDs. If conservative treatment fails, surgical intervention may be required. Even after successful surgery, these procedures often result in reduced joint mobility and tendon or ligament strength.

A novel flowable tissue matrix allograft, derived from human placental connective tissue, has recently been made available for minimally invasive treatment of damaged or inadequate tissue  (PX50®, Human Regenerative Technologies LLC, Redondo Beach, CA).

Based on the universal role of connective tissue in the body, and its reported antimicrobial, anti-adhesive, and anti-inflammatory properties, we assessed the effects of using this placental tissue matrix in the treatment of a series of lower extremity injuries.

In this pilot study, 9 of 10 patients reported pain levels of 2 or less by week four using the VAS pain scale. This short-term pilot study effectively shows that injectable, flowable amniotic allografts can be used for orthopedic sports injuries of the lower extremities.

## Introduction

Damaged connective tissue commonly leads to lower extremity injuries. These injuries can result in inflammation, reduced mobility, and chronic pain [1]. Conservative treatment may include orthotics, offloading the injury, physical therapy, NSAIDs. If conservative treatment fails, surgical intervention may be required. Even after successful surgery, these procedures often result in reduced joint mobility and tendon or ligament strength [2-3].

A novel flowable tissue matrix allograft, derived from human placental connective tissue, has recently been made available for minimally invasive treatment of damaged or inadequate tissue  (PX50®, Human Regenerative Technologies LLC, Redondo Beach, CA).

Based on the universal role of connective tissue in the body [4], and its reported antimicrobial, anti-adhesive, anti-inflammatory properties, we assessed the effects of using this placental tissue matrix in the treatment of a series of lower extremity injuries.

### Characteristics of connective tissue

Connective tissue is one of the four fundamental types of biological tissue found in the human body [4]. It is the most abundant, widely distributed tissue [4], making up a large proportion of the total body mass [5].

Connective tissue is found under the skin and all epithelia; in the outer covering of nerves; around organs; in the fascia between muscles; in the fibrous sheath around bones; and creates tendons, ligaments, cartilage and bones [4].

All connective tissue consists of three main components: Fibers, Ground Substance and Cells. The cells of connective tissue are embedded in an ExtraCellular Matrix (ECM). The ECM, in turn, consists of the fibers embedded in a featureless ‘ground substance’ [5].

The function of connective tissue in the body includes the binding of organs, structural support, physical protection, immune protection, and movement [4].

### The placenta as a source of connective tissue

The human placenta is a potentially rich source of biological tissue, comprising the placenta, the amniotic membranes, the amniotic fluid, the umbilical cord, and the umbilical cord blood [6].

The extracellular matrix (ECM) components of these tissues include collagens (Types I, III, IV, V, and VI), fibronectin, nidogen, laminin, proteoglycans, and hyaluronan, as well as growth factors [45].

Placental tissues have been reported to be non-immunogenic, providing a tissue source that elicits little or no immune response in the patient, reducing the risk of rejection, and graft failure [10-14]. Further, these tissues have been shown to be anti-microbial, regulate inflammation, prevent fibrosis, and support correct tissue reconstruction.

### History of clinical use

Historically, most clinical reports on the use of placental tissues have discussed the use of amniotic membranes for an array of clinical applications since the 1920’s [15-19]. These uses include general surgery [20-21], corneal surgery [22-24], plastic surgery [25], burns and wound care [26-32], sports medicine [33-34], foot and ankle procedures [35-36], spine and dura repair [37-44], nerve wrap or dural covering [39, 42, 45], and tendon repair [46].

## Materials and methods

The investigator contracted with Midlands IRB for this study. Midlands IRB ruled that this pilot retrospective study review of existing patient records was exempt from IRB review in accordance with 45CFR46.101B. The data collected was without identifiers or link to identifiers. Additionally, the investigator who would normally have access to the records as part of the patients’ routine clinical care conducted the records review. Patients were consented to treatment prior to engagement of the injection protocol.

A retrospective pilot cohort study of 10 patients were selected from the treating physician’s own patient population at a single clinical site. Male and female patients were randomly selected without bias to gender. Patients were identified as having a musculoskeletal injury involving the lower extremity. All patients were initially screened using the Visual Analog Pain Scale. Patients were identified with either acute or chronic tendon or muscular injuries of the lower extremities, including: posterior tibial tendonitis, peroneal tendonitis, anterior tibial tendonitis, extensor muscles of the foot,  plantar musculature of the foot excluding the plantar fascia, and Achilles tendonitis.

Exclusion criteria included joint arthritis, plantar fasciitis, cellulitis of the lower leg, infection of the lower leg, or hypersensitivity to amniotic tissue products.

The qualifying patients were then identified as having the acute or chronic tendon injury defined through history and physical and musculoskeletal advanced ultrasound imaging by the treating physician. Visual pain scales were used on a weekly basis to assess patient’s self-reported pain level starting at week 0 and followed through to week six. 

Ultrasound non-vascular extremity examinations were performed at week 0 at the time of injection of the flowable allograft tissue matrix and again at week four and week six. Images were recorded in real-time and assessed in both longitudinal and transverse planes. 

The investigator on every patient performed the injection technique of the allograft tissue. The technique included a mixture of 1.0 cc Bupivicaine 0.5% plain and 0.5 cc of the ambient temperature, flowable allograft tissue matrix. The injection was performed under a sterile field condition using ultrasound guidance to target the injection material to the area of injury. 

Patients were then dispensed a pneumatic short leg ankle walking boot (Ossur) and instructed to utilize the boot for weight-bearing for two weeks post-injection. Patients were instructed how to use the walking boot properly and effectively for off-loading and stabilization post-injection.

In addition, demographic, injury history, and previous treatments (if applicable) were obtained. This included age, gender, ethnicity, smoking history, and BMI. 

## Results

Table 1 summarizes patient characteristics. 

**Table 1:** Demographic Data

Patient	Age	Gender	BMI
1	63	F	31.12
2	62	F	37.32
3	35	M	26.41
4	77	M	36.82
5	23	F	15.55
6	54	F	25.56
7	34	F	17.42
8	45	F	28.84
9	61	F	33.51
10	51	M	26.74

Age ranges were from 23 to 77 with a median of 52.5. Seven patients were female and three were male. Almost all (nine) of the patients were white, non-Hispanic. BMI ranged from 15.55 to 37.32 with a median of 27.79. Posterior tibialis tendonitis was the predominant injury treated (n=4), followed by Achilles tendonitis (n=2), flexor hallucis tendonitis (n=2), anterior tibialis tendonitis (n=1), and abductor digiti minimi tendonitis (n=1). 

Table 2 summarizes the observations of VAS pain scores at each time point per patient.

**Table 2:** VAS Pain Scores by Visit

	Week 0	Week 1	Week 2	Week 3	Week 4	Week 5	Diagnosis		
1	7	1	0	0	0	0	FHL tendonitis right		
2	7	3	3	2	0	0	Achilles tendonitis left		
3	9	1	1	1	0	0	Achilles tendonitis left		
4	8	2	1	1	0	0	Abductor digiti quinti left		
5	8	2	2	1	1	0	Post tibialis tendonitis left		
6	7	4	9	9	2	0	Post tibialis tendonitis right		
7	8	2	2	0	0	0	Post tibialis tendonitis right		
8	2	3	1	1	0	0	Post tibialis tendonitis right		
9	8	2	1	1	0	0	Ant tibialis tendonits left		
10	10	2	1	1	0	0	FHL tendonitis right		

Overall, all 10 patients improved to pain scores of 0 by the end of the evaluation period. Nine of the 10 patients declined as expected with their pain ratings. Only one patient had an increase of pain, and that was due to another area of the posterior tibialis tendon becoming pathologic outside of the injected target area. By week three, in nine of the 10 patients enrolled, all had pain levels improved 75% or greater by week three.

Eight of the 10 patients reported VAS pain score of 0 by week 4.

**Figure 1:**
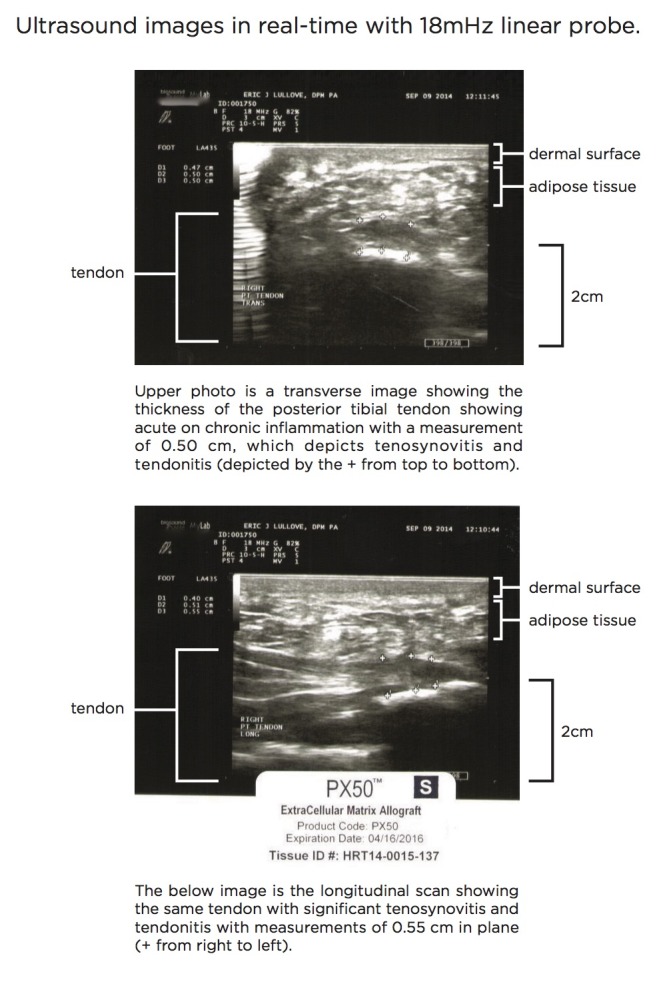
Pre-Injection Ultrasound Posterior Tibialis Tendon The ultrasound scan depicted shows both transverse (top) and longitudinal (bottom) planes of imaging. In the upper image, the measurement of the tendon (depicted by the hypoechoic signal between the fascial planes near the bottom) shows 0.50 cm in diameter, reflecting a moderately inflammed tendon. The bottom image reflects the same measurement (the hypoechoic signal between the fascial planes in the middle of the image) depicting the inflammatory tendonitis of overuse in this patient selected for injection.

Pre-injection pathology is shown in Figure 1.

**Figure 2:**
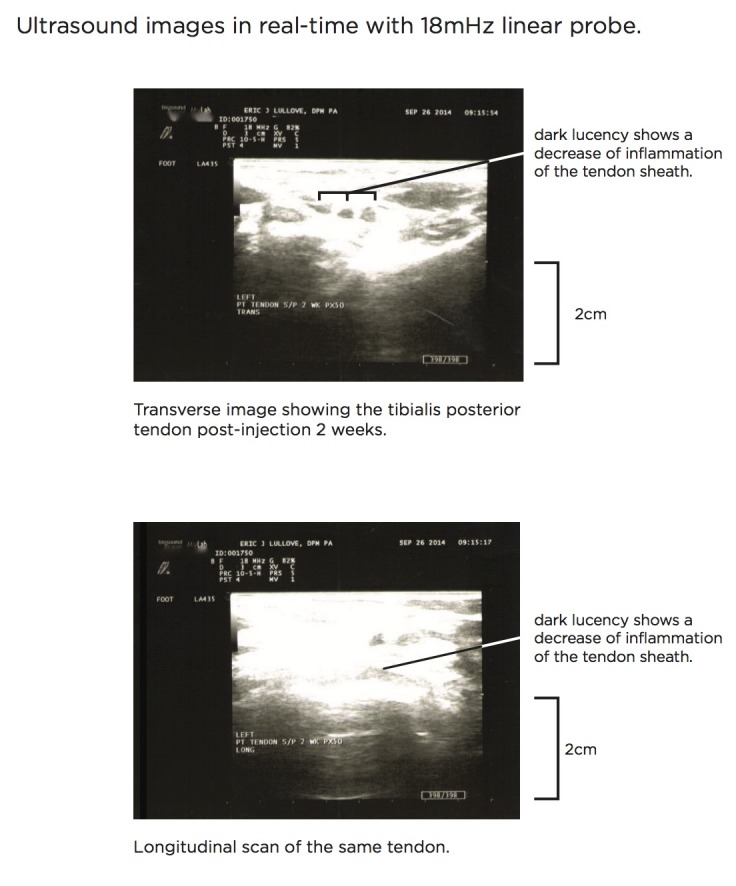
Post-Injection Ultrasound Posterior Tibialis Tendon The ultrasound scan depicted shows both transverse (top) and longitudinal (bottom) planes of imaging. In the upper image, the measurement of the tendon two weeks post-injection shows a measurement of 0.21 cm, which is a normal tendon measurement. The bottom image shows a measurement of 0.24 cm, which also reflects a normal tendon measurement. This reflects a 90% to 95% reduction in inflammation and a good therapeutic response post-injection.

Figure 2 shows post-injection pathology at two weeks.

## Discussion

Overall, all ten patients resolved their pain by week five. Based on the VAS pain scores reported, these 10 patients show a strong correlation for using amniotic tissue allograft injection to resolve acute tendon injuries rapidly.  Further research would be needed to compare the use of amniotic allograft tissue injections versus corticosteroid injections head-to-head, and further larger studies, including randomized controlled trials, may elucidate the reasons for these differences.

In patients with acute tendon or musculoskeletal injuries of the lower extremities, the use of this newer injectable amniotic tissue allograft shows efficacy in reduction of pain within six weeks of injection. This particular therapy shows extensive promise in the ability to keep patients adherent to treatment regimens with a higher retention rate of success than standard tendon injury treatment techniques. 

In most cases of acute on chronic tendon injury, the need for regenerative medicine modalities only increases as the population increases in age and activity level. With the advent of more active older adult lifestyles, the need for these types of therapies will be more in-demand than in years past. As technologies advance with better sports gear and equipment, so must our need as physicians to improve the outcomes of patients whose motives are for a quicker return to activities without the need for advanced surgical treatments. The need for advanced medical alternatives to surgery must be paramount in the treatment of these patient populations now and in the future.

## Conclusions

Due to the nature of this single-center, single investigator study, further research is needed with a larger sample of patients to verify the VAS pain score ratings encountered here in this pilot study. While there was bias from a single investigator, the author was very careful with regard to moderating the scoring system used by the patients to report their pain. Also, the author made every attempt to reduce bias by standardizing the injection technique, the administration of local anesthetic in similar amounts to each patient, and the utilization and interpretation of ultrasound guidance.

Due to the nature of only a few injectable amniotic tissue allografts in the market, it is necessary for qualified health professionals choose the proper therapy for patients with acute tendon injuries. As with all injectable treatments, the standards of care should be used in conjunction with newer allograft techniques to obtain optimal results.
